# Development and Optimization of CRISPR Prime Editing System in Photoautotrophic Cells

**DOI:** 10.3390/molecules27061758

**Published:** 2022-03-08

**Authors:** Zhengzheng Jiang, Abdullah, Shun Zhang, Yuli Jiang, Rui Liu, Yi Xiao

**Affiliations:** 1 State Key Laboratory of Microbial Metabolism, School of Life Sciences and Biotechnology, Shanghai Jiao Tong University, 800 Dongchuan RD. Minhang District, Shanghai 200240, China; jiangzhengzheng@sjtu.edu.cn (Z.J.); abdullaa@sjtu.edu.cn (A.); zhangshun@sjtu.edu.cn (S.Z.); yuli.jiang@leica-microsystems.com (Y.J.); liu24110@sjtu.edu.cn (R.L.); 2 Joint International Research Laboratory of Metabolic & Developmental Sciences, Shanghai Jiao Tong University, 800 Dongchuan RD. Minhang District, Shanghai 200240, China

**Keywords:** prime editor, *Arabidopsis*, nCas9(H840A), indel, microalgae

## Abstract

Prime editor (PE), a versatile editor that allows the insertion and deletion of arbitrary sequences, and all 12-point mutations without double-strand breaks (DSB) and a donor template, dramatically enhances research capabilities. PE combines nickase Cas9(H840A) and reverse transcriptase (RT), along with prime editing guide RNA (pegRNA). It has been reported in several plant species, but a weak editing efficiency has led to a decrease in applications. This study reports an optimized-prime editor (O-PE) for endogenous gene editing in *Arabidopsis thaliana* cells, with an average 1.15% editing efficiency, which is 16.4-fold higher than previously reported. Meanwhile, we observed an increase in indels when testing alternative reverse transcriptase and found out that nCas9(H840A) fused to non-functional reverse transcriptase was responsible for the increase. This work develops an efficient prime editor for plant cells and provides a blueprint for applying PE in other photoautotrophic cells, such as microalgae, that have a high industrial value.

## 1. Introduction

The development of facile, precise, and efficient genetic editors is essential for engineering organisms. The Clustered Regularly Interspaced Short Palindromic Repeats-CRISPR associated protein (CRISPR-Cas) system, which naturally evolved as a prokaryotic defense system has been extensively exploited to develop editing tools by combining it with cell’s repair mechanisms [1]. The early CRISPR-based editing tools relied heavily on the ability of Cas protein to introduce a double-strand break (DSB) at the target site. This was followed by the introduction of a donor template, along with a reliance on cell repair machinery, either homologues repair (HR) or non-homologues end-joining (NHEJ), for insertion or deletion of the sequence [2]. To counter the issues of DSB-based editors, base editing systems have been developed, which allow DSB-free and template-free editing [3,4]. However, base editors are restricted to base substitutions. There remains a gap for editors that allow DSB-free and template-free insertions, deletions, transition, and transversion; in short, an all-round editor.

**Figure 1:**
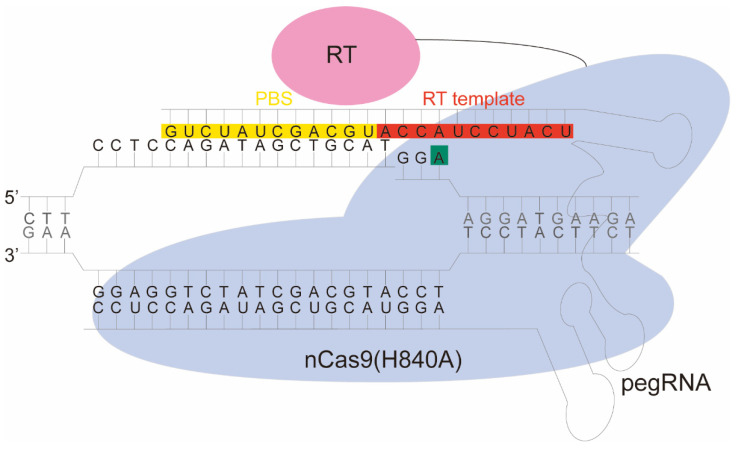
Illustration of PE system. Prime editor consists of nCas9(H840A) fused to reverse transcriptase (RT) via XTEN linker (a 16 amino acid flexible linker, black line). The fusion protein is guided by a pegRNA which consists of primer binding site (PBS), RT template and sgRNA. PBS (yellow), RT template (red). This figure shows how a mutation (+3 A to T) was introduced (green).

Recently, a versatile editing tool, i.e., prime editor (PE), was reported, allowing insertion and deletion of an arbitrary sequence and all 12-point mutations, without DSB and a donor template [5]. PE consists of reverse transcriptase fused to nickase Cas9(H840A) via a flexible linker (nCas9-RT) (Figure 1). The nCas9-RT uses a uniquely designed prime editing guide RNA (pegRNA). The pegRNA contains a single guide RNA (sgRNA) along with primer binding site (PBS) and reverse transcriptase template (RT template), which carries the desired edits. Upon prime editing, nCas9 nicks the non-target strand, PBS binds to the 3′flap acting as a primer for RT, and transcribes the RT template containing the desired edit. The flap is resolved during DNA repair and replication, and the edit is permanently inserted into the target site. The first kind of PE, i.e., PE1, was optimized by developing an engineered M-MLV RT (D200N + L603W + T330P + T306K + W313F); thus, creating the PE2 system. PE2 led to a 1.6 to 5.1-fold increase in point mutation efficiency over PE1. PE2 was further engineered to develop PE3, which involved a second cleavage at an unedited strand 14 to 116 bases away from the original nick. This led to a further 4-fold increase in editing efficiency over PE2. A tool of such versatility holds great promise for plant genetic engineering. Prime editors have been successfully applied to higher plant cells such as rice [6,7,8,9,10,11,12,13], wheat [6], maize [14], tomato [15], tobacco [13], and *Arabidopsis* [13].

Here, we report the development of PE2 for *Arabidopsis* cells. The weak PE efficiency led us to optimize it to enhance editing efficiencies. We tested multiple combinations of promoters and reverse transcriptase. Our optimized-PE2 (O-PE2) system led to precise endogenous gene editing in *Arabidopsis,* with an average 1.15% editing efficiency, an 8.7-fold increase in editing efficiency compared to unoptimized-PE2 (pPE2-35Srat), which was 16.4-fold higher than a previous study [13]. Moreover, when testing alternative reverse transcriptase, we observed a rise in indels with PE2. After investigating the cause, we concluded that nCas9(H840A) fused to a non-functional RT was the culprit. Our strategy provides a blueprint for the development and evolution of PE into photoautotrophic microalgae, whose development of various value-added industrial and pharmaceutically active substances is hindered due to a limited molecular toolbox [16].

## 2. Results

### 2.1. Development of Prime Editing in E. coli

**Figure 2:**
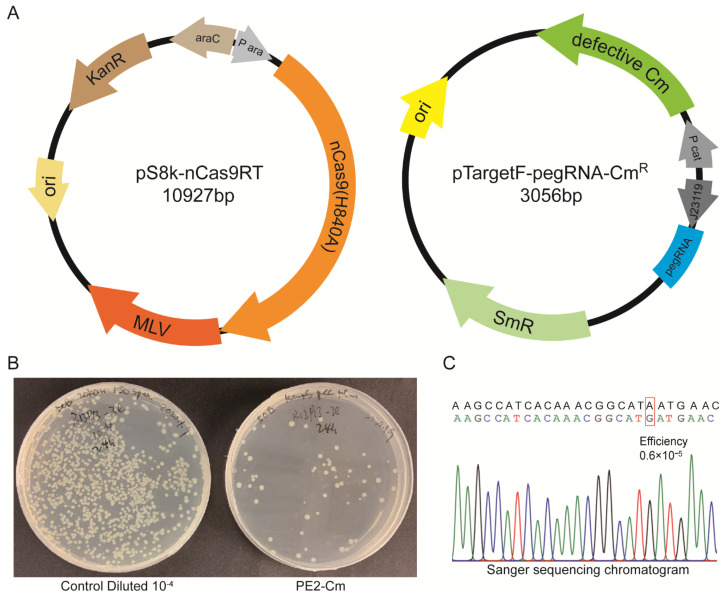
Prime editing in *E. coli*. (**A**) Diagram of the PE2 system plasmids in *E. coli*. The pS8k-nCas9RT consists of nCas9(H840A) fused to M-MLV RT induced by arabinose inducible promoter and Kan resistance. The pTargetF-pegRNA-Cm^R^ consists of dysfunctional Cm^R^ H139Y, pegRNA targeting Cm^R^ H139Y expressed via constitutive *J23119* promoter, and spectinomycin resistance gene. (**B**) Dual-plasmid transformed *E. coli* MG1655 was serially diluted and plated. Control diluted 10^−4^ on kan + spec plate (left), recovered Cm^R^ via PE2 on kan + spec + cm plate. (**C**) A sanger sequencing chromatogram and editing efficiency.

To verify the prime editing system, we initially developed a prime editor for *E. coli*, following the reported design in yeast and mammalian cells [5] (Figure 1). We first used a BglBrick Ps8K plasmid [17] to construct the PE2 system, consisting of arabinose-induced nCas9(H840A) fused to M-MLV RT at C-terminus via a flexible linker (Figure 1 and Figure 2A). Then, a second plasmid pTargetF-pegRNA-Cm^R^ was constructed based on pTargetF [3], consisting of a dysfunction chloramphenicol resistance gene Cm^R^ H139Y and constitutively expressed pegRNA targeting Cm^R^ H139Y (Figure 2A). The H139Y mutation renders Cm^R^ non-functional, while PE2 can revert the mutation, making Cm^R^ functional.

We carried out a two-plasmid transformation in electrocompetent *E. coli* MG1655 (Figure 2B), and the cells were then grown on an agar plate, with or without chloramphenicol. As mentioned in the methods, the editing efficiency was calculated and confirmed by Sanger sequencing. Editing via PE occurred and rendered the mutants chloramphenicol resistance, but the editing efficiency was extremely low up to 0.6 × 10^−5^ (Figure 2C). The conversion of A to G in Cm^R^ H139Y gene from the strains with chloramphenicol resistance was identified by sequencing (Figure 2C), confirming our PE is functional in *E. coli.*

### 2.2. Development of PE2 in Arabidopsis Cells

To develop PE2 for *Arabidopsis* cells, we constructed a pPE2-35SRat plasmid, based on pBbE1 [17] plasmid. The pPE2-35SRat plasmid consists of codon-optimized nCas9(H840A)-M-MLV RT fused to green fluorescence protein via T2A linker [18] and pegRNA expressed constitutively by *Arabidopsis* codon-optimized *U6* promoter (Its sequence is shown in Supplementary Materials). The pegRNA targeted *PDS3*, which is important for dwarfism and mosaic albino phenotype [19], for +3 A to T base substitution (Figure 1).

**Figure 3:**
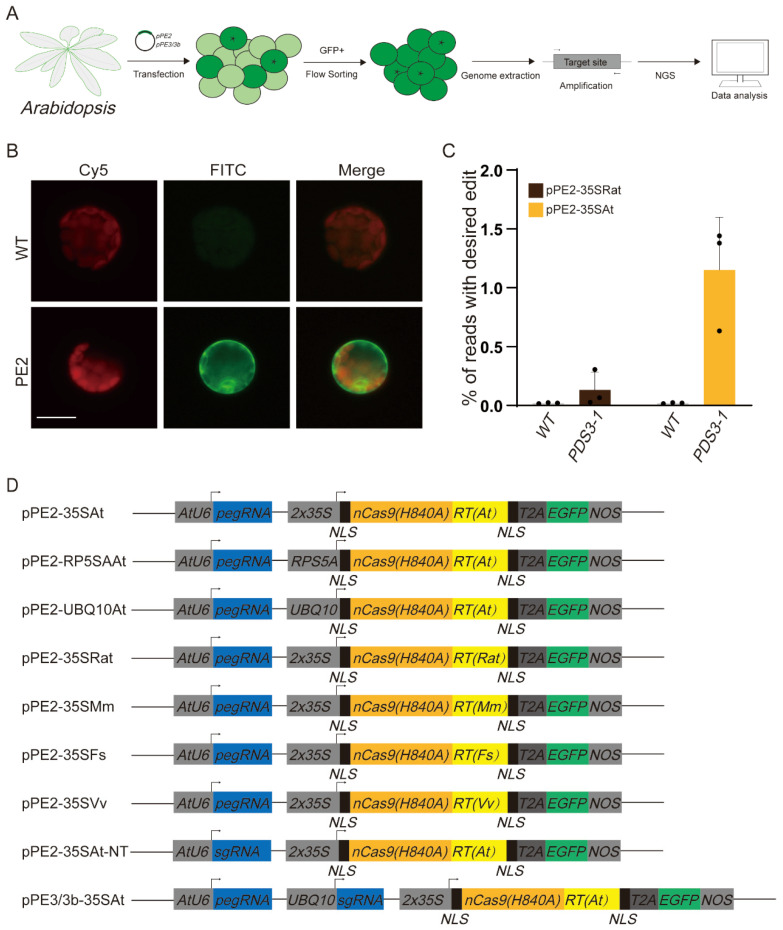
Prime editing in *Arabidopsis* cells. (**A**) The *Arabidopsis* cells were transfected with the PE system. The positively transfected cells were selected via flow cytometry. This was followed by genome extraction, amplification of target sequence, NGS, and data analysis. (**B**) Fluorescence of GFP+ protoplasts. Red signal in Cy5 channel indicates autofluorescence from chloroplast, green signal indicates GFP protein overexpressed in the cytoplasm from plasmids. Untreated protoplast served as a negative control. Scale bar, 20 µm. (**C**) Frequency reads with desired edits (%) of pPE2-35SRat and pPE2-35SAt at *PDS3-1* site using next-generation sequencing (NGS). Black balls indicate independent results. Error bars represent mean ± s.d. of n = 3 independent biological replicates. (**D**) Features of each vector in *Arabidopsis* cells.

To test the constructed PE2 in *Arabidopsis* cells, we transformed the pPE2-35SRat plasmid into *Arabidopsis*. We selected 3 to 4 weeks old *Arabidopsis* leaves and processed them into protoplasts and then transfected the pPE2-35SRat plasmid into them using a PEG transfection buffer. The positively transfected cells presented green fluoresce (Figure 3B), owing to the *GFP* gene in the plasmid. The transformed cells were selected and collected by flow cytometry, based on GFP fluoresces. The collected cells were then used for genome extraction, and PCR amplified the target sequences of *PDS3-1*. The amplicons were sequenced by next-generation sequencing and analyzed using CRISPResso2 [20]. The research scheme is shown in Figure 3A. The results showed a base substitution efficiency of 0.1329% (Figure 3C and Table 1), similar to the previous report [13]. However, the PE2 system was less efficient in *Arabidopsis* compared with other higher plants such as rice.

### 2.3. Optimization of PE2 in Arabidopsis Cells

Considering the weak PE2 efficiency, we tweaked the system using various promoters for the fusion protein and RTs. We tested *2x35S*, *RPS5A,* and *UBQ10* promoters, common promoters for protein expression in *Arabidopsis* cells. We also tested different RTs: *Fusicatenibacter saccharivorans*-RT [21] (Fs-RT), *Marinomonas mediterranea*-RT [22] (Mm-RT), *Vibrio vulnificus*-RT [23] (Vv-RT), and *Arabidopsis* codon-optimized-RT. All the sequences are shown in the Supplementary Materials. We constructed total of six plasmids based on these, named pPE2-35SAt, pPE2-RPS5AAt, pPE2-UBQ10At, pPE2-35SFs, pPE2-35SMm, and pPE2-35SVv (Figure 3D). These plasmids were individually introduced into the protoplasts for the PE editing test through the method mentioned earlier. Finally, we successfully obtained a highest efficiency of 1.44% (based on three independent experiments, the efficiencies were 1.44%, 1.38%, and 0.63%, respectively) by combining the *35S* promoter and *Arabidopsis* codon-optimized reverse transcriptase (Figure 3C,D and Table 1), namely optimized-PE2 (O-PE2). The others did not work well, with their efficiencies ranging between 0.0187% and 0.0342% (Table 1). Compared to initially constructed pPE2-35SRat, pPE2-35SAt (O-PE2) boosted PE2 efficiency to 8.7-fold at *PDS3-1* loci.

Based on the O-PE2 system, we developed a PE3/3b system (pPE3-35SAt/pPE3b-35SAt Figure 3D) in *Arabidopsis* cells. To our surprise, contrary to reports in other species and mammalian cells [5], PE3/3b did not significantly improve the modification efficiency, indicating that the addition of another sgRNA does not improve the efficiency in *Arabidopsis* cells in the current conditions.

### 2.4. nCas9(H840A) in PE2 Cause Indels

**Figure 4:**
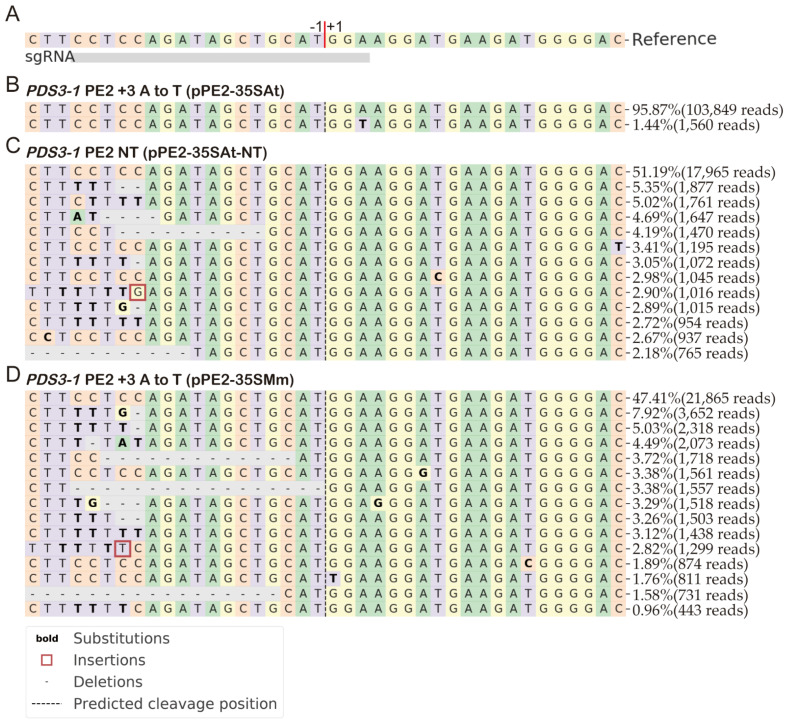
NGS analyzed data. Alleles of *PDS3-1* site isolated from protoplasts of *Arabidopsis* after editing with pPE2-35SAt, pPE2-35SAt-NT, and pPE2-35SMm. The amplicons sequenced by an Illumina MiSeq for NGS were analyzed using CRISPResso2. (**A**) represents the wild-type reference sequence. (**B**) represents the successful PE2 via pPE2-35SAt. (**C**) represents control consisting of sgRNA and not pegRNA. (**D**) represents indels when pPE2-35SMm was used.

While testing alternate promoter/reverse-transcriptase combinations, we observed an increased variation in the DNA sequence at the target site with some combinations (Table 1 and Figure 4). The PE plasmids, pPE2-RPS5AAt, pPE2-UBQ10At, pPE2-35SFs, pPE2-35SMm, and pPE2-35SVv did not perform the desired editing. The sequencing results also showed a lower proportion of wild-type sequence reads between 40.36% to 84.56%, contrary to O-PE2, which had 94.23% wild-type reads. The altered reads included insertions, deletions, or substitutions near the target sequence. We hypothesized that in those cases, reverse transcriptase was non-functional, leaving the nCas9(H840A) cut to be repaired, which led to indels during the repair process. To confirm this hypothesis, we constructed pPE2-35SAt-NT, which lacks PBS and a RT template targeting the *PDS3-1* locus. The NGS results showed only 50.73% of reads were the wild-type sequence, while the rest contained indels (Figure 4 and Table 1). These results supported the hypothesis that indels were observed when RT was non-functional. At *PDS3-2*, where PE2 worked, we observed negligible indels, indicating that indels are only caused when RT is non-functional (Table 1).

## 3. Discussion

CRISPR-Cas9-mediated gene-editing tools, including PE, have been widely applied in plant cells [24], to increase yield, regulate metabolites, improve stress resistance, etc. [25]. However, the editing efficiency of the PE system is still relatively lower than that of the traditional Cas9 editing tools. The editing efficiency of the PE system in different species is affected [26] by the selection of the spacer position, length of the PBS, size of the RT template, and their combination [5,6,27].

A previous report of the development of PE for *Arabidopsis* involved the insertion of *GFP11* at the *AT1G26660.1* locus, but reported an extremely low editing efficiency of 0.07 ± 0.12% [13]. In this paper, we improved the editing efficiency of PE in *Arabidopsis,* by optimizing combinations of different promoters and reverse-transcriptase. We used *35S* promoter and *Arabidopsis* codon-optimized RT to achieve an average 1.15% single base substitution modification efficiency at the endogenous gene, for the first time. Further optimization and improvement of the editing efficiency of O-PE could involve adjusting pegRNA by following the guidelines from the research of the Gao group [26] and Liu group [28]. In addition, we tested PE3/3b in *Arabidopsis* and, consistent with previous studies [6], found no significant improvement in editing efficiency.

Furthermore, we found that nCas9(H840A) led to indels during the testing of alternate promoters and reverse-transcriptase. Previous work from the Liu group [29] reported the same observation in the base editor, in which nCas9(H840A), and not nCas9(D10A), caused a high proportion of indels in animal cells. However, this was random and altered with the target site. This study designed a plasmid (pPE2-35SAt-NT) as a control, which carried sgRNA. The analysis of NGS showed that at sites where PE2 was efficient, nCas9(H840A) caused a large number of indels when fused to dysfunctional reverse-transcriptase. Contrarily, when the reverse-transcriptase was functional, PE2 did not cause indels.

Adoption of PE is an important addition to the molecular toolbox and goes beyond *Arabidopsis*. Microalgae are considered a third-generation biofuel. It has incredibly high bioenergy, aquatic products, food, pharmaceutical, and medical value. The traditional method to improve the yield of microalgae is to control the accumulation of different metabolites by regulating the carbon flow, by changing the culture conditions, such as pH, temperature, and nitrogen stress. However, such methods have certain limitations that affect cell division and ultimately reduce the overall biological yield [30]. The development of versatile and efficient molecular tools can overcome such drawbacks, by overexpression or repression of desired pathways via genetic manipulation. CRISPR-Cas based tools have been deployed in *Chlamydomonas reinhardtii* [31]. However, the dependence of previous editors on DSB, homologous recombination, and the need for template DNA restricts their application. As a new generation of gene editors, PE has incomparable advantages over the previous generation and is an excellent candidate for gene editing of microalgae. To date, there have been no reports of prime editing in microalgae.

In summary, we developed an optimized-PE2 (O-PE) editor for *Arabidopsis*, achieving an average substitution efficiency of 1.15% of endogenous genes. We further investigated the indel formation in cases where RT was dysfunctional and proved nCas9(H840A) to be the main factor. Our strategy of constructing PE editors via a combination of various promoters, RT, and sequence codon-optimization provides a blueprint for the development of PE for genetically engineered high-yield microalgae [16,30,32].

## 4. Materials and Methods

### 4.1. Plasmid Construction

To construct vector pPE2-35SRat, NLS, *U6* promoter, double *35S* promoter, and nCas9(H840A) codon-optimized for *Arabidopsis* by Tsingke, Shanghai, China, the engineered M-MLV reverse transcriptase which was amplified from Addgene# 132775, Cambridge MA, USA, EGFP which was from Prof. Gong, and T2A linker were cloned into the vector pBbE1 [17] backbone, yielding the various nCas9(H840A)-RT fusion plasmids by Golden Gate. AT-RT were codon-optimized for *Arabidopsis* by Tsingke. The *UBQ10* promoter was a gift from Prof. Gong. The natural reverse transcriptase-Cas1 fusion protein Mm-RT [22], Vv-RT [23] and Fs-RT [21] were synthesized by Genewiz, Shanghai, China. The pegRNAs were created by paired primers containing the target sgRNA, PBS, and RT template sequences using T4 ligase (NEB) and introduced into the vectors by Golden Gate. Another sgRNA of PE3/PE3b used the same method for introduction into vectors. All the plasmid constructions were confirmed by clone PCR, enzyme digest, and Sanger sequencing.

The *E. coli* PE system, i.e., pS8k-nCas9RT, consisted of a low copy Sc101 ori and kanamycin resistance gene cloned with arabinose-induced nickase Cas9(H840A) fused to engineered M-MLV reverse-transcriptase via XTEN linker [5]. The second plasmid pTargetF-pegRNA-CmR consisted of high copy colE1 ori, spectinomycin resistance gene, dysfunctioned chloramphenicol resistance gene Cm^R^ H193Y, and pegRNA expressed constitutively by *J23119* promoter.

### 4.2. Efficiency Assay in E. coli

For PE assay in *E. coli*, we performed dual-plasmid transformation of pS8k-nCas9RT and pTargetF-pegRNA-Cm^R^ to electric component *E. coli* MG1655 cells. After transformation, cells were recovered for 1 h at 37 °C at 200 rpm. The recovered cells were transferred to 5 mL fresh LB and inoculated overnight at 37 °C at 220 rpm. The cells were then serially diluted and plated on kanamycin (Kan) + Spectinomycin (Spec) and kanamycin (Kan) + Spectinomycin (Spec) + Chloramphenicol (Cm) plates. The editing efficiency was calculated as follows:

$$\frac{\mathrm{No}.\;\mathrm{of}\;\mathrm{clones}\;\mathrm{on}\;\mathrm{Kan}\;+\;\mathrm{Spec}\;+\;\mathrm{Cm}\;\mathrm{plate}}{\mathrm{No}.\;\mathrm{of}\;\mathrm{clones}\;\mathrm{on}\;\mathrm{Kan}\;+\;\mathrm{Spec}\;\mathrm{plate}}$$

### 4.3. Protoplast Transfection

Plants were grown at 22 °C under 12 h light and 12 h dark cycles. Three to four-week-old Col-0 leaves were used for the protoplast transient assay, and the protocol was from Sheen Lab [33]. We isolated protoplasts from leaf strips using an enzyme solution (1.4% cellulose (Yakult, Nagoya, Japan), 0.32% macerozyme (Yakult), 0.4 M mannitol (Sigma, Burlington, VT, USA), 20 mM KCl (Sinopharm, Shanghai, China), 20 mM MES (4-Morpholineethanesulfonic acid hydrate, 2-(N-Morpholino) ethanesulfonic acid hydrate, Sigma), pH 5.7, 10 mM CaCl_2_ (Sinopharm), 0.1% BSA (bovine serum albumin, Sigma)), which was shaken (20 rpm) in the dark at room temperature for 2 h. The digest reaction was terminated by W5 solution (2 mM MES (pH 5.7) (Sigma), 154 mM NaCl (Sinopharm), 125 mM CaCl_2_ (Sinopharm), 5 mM KCl (Sinopharm)) and filtered through a 70-μm cell strainer (Falcon, New York, NY, USA). After removing undigested cellular debris, protoplasts were centrifuged (1400 rpm) and resuspended with pre-chilled W5 solution twice. The protoplasts were placed in 5 mL W5 solution in ice for at least 0.5 h, following washing steps. Then, after removing the supernatant, cells were quantified using a hemocytometer and resuspended in MMG solution (4 mM MES (pH 5.7) (Sigma), 0.4 M mannitol (Sigma), and 15 mM MgCl_2_ (Sinopharm)). The plasmids (10 μg) were introduced into 2 × 10^5^ cells (100 μL) using 1.1-fold volume PEG transfection buffer (40% PEG4000 (poly ethylene glycol, Sigma), 0.4 M mannitol (Sigma), and 0.1 M CaCl_2_ (Sinopharm)). The transfections were terminated with 5-fold volume W5 solution and then centrifuged (100 g). The cells were resuspended with 250 μL WI solution (4 mM MES (pH 5.7) (Sigma), 0.5 M mannitol (Sigma), 20 mM KCl (Sinopharm)) into 24-well plates for 16 h in the dark, before microscopy assay or FACS (Fluorescence activated Cell Sorting) separation.

### 4.4. DNA Extraction and Amplicon Sequencing

Genomic DNA of protoplasts was extracted using a DNAsecure Plant Kit (Tiangen, Shanghai, China), and Target sequences were amplified using Phusion polymerase (Thermo Fisher Scientific, Waltham, MA, USA) with specific primers (Supplementary Materials) for Sanger sequencing (Tsingke, Shanghai, China) or next-generation sequencing (Sangon/Biozeron, Shanghai, China). NGS reads were analyzed using CRISPResso2 (version 2.0.38; Cambridge, MA, USA, 2019) [20].

## Figures and Tables

**Table 1:** Efficiencies of various prime editors in protoplasts of *Arabidopsis thaliana*.

Plasmid	Target Site	Aim	Average Efficiency	Average Proportion of NGS Reads with No Changes (%)
Non ^1^	*PDS3-1*	/	0.0203%	90
pPE2-35SAt	*PDS3-1*	+3 A to T	1.1506%	94.23
pPE2-RPS5AAt	*PDS3-1*	+3 A to T	0.0342%	58.05
pPE2-UBQ10At	*PDS3-1*	+3 A to T	0.0194%	77.89
pPE3b-35SAt	*PDS3-1*	+3 A to T	0.0318%	78.42
pPE2-35SRat	*PDS3-1*	+3 A to T	0.1329%	86.39
pPE2-35SFs	*PDS3-1*	+3 A to T	0.0255%	84.56
pPE2-35SMm	*PDS3-1*	+3 A to T	0.0214%	49.56
pPE2-35SVv	*PDS3-1*	+3 A to T	0.0187%	40.36
pPE2-35SAt-NT	*PDS3-1*	/	0.0336%	50.73
Non ^1^	*PDS3-2*	/	0.0997%	93.39
pPE2-35SAt	*PDS3-2*	+5 G to A	0.1167%	92.83
Non ^1^	*ALS-1*	/	0.1369%	93.47
pPE3-35SAt	*ALS-1*	+2 A to G	0.1547%	91.71

^1^ No vectors.

## Data Availability

Data available from the corresponding author: yi_xiao@sjtu.edu.cn.

## Supplementary Materials

The supporting Sequence information can be downloaded at https://www.mdpi.com/article/10.3390/molecules27061758/s1.

## Sample Availability

Samples from the compounds are available from the corresponding author: yi_xiao@sjtu.edu.cn.
