# Supplementary material for: Development and Optimization of CRISPR Prime Editing System in Photoautotrophic Cells
Source: Molecules. 2022 Mar 8;27(6):1758. doi: 10.3390/molecules27061758 (PMC8948812; doi:10.3390/molecules27061758)
Supplement: Supplementary file 1 [file molecules-27-01758-s001.zip › molecules-1626106-supplementary.pdf]

Supplementary Sequences Information：

Development and optimization of CRISPR prime editing system in photoautotrophic cells

Zhengzheng Jiang[1], Abdullah[1], Shun Zhang[1], Yuli Jiang[1], Rui Liu[1], Yi Xiao[1,2]

1 State Key Laboratory of Microbial Metabolism, School of Life Sciences and Biotechnology, Shanghai Jiao Tong University, 800 Dongchuan RD. Minhang District, Shanghai 200240, China; E-mail: yi_xiao@sjtu.edu.cn(Y.X.)

2 Joint International Research Laboratory of Metabolic & Developmental Sciences， Shanghai Jiao Tong University, 800 Dongchuan RD. Minhang District, Shanghai 200240, China

1. Sequences of primary PE2 used in this study

pPE2-35SRat：

CCAAAGAAAAAGAGAAAGTAGATAAGAAGTACTCTATCGGACTCGACATCGGAACTAACTCTGTGGGATGGGCTGTGAT
CACCGATGAGTACAAGGTGCCATCTAAGAAGTTCAAGGTTCTCGGAAACACCGATAGGCACTCTATCAAGAAAAACCTTAT
CGGTGCTCTCCTCTTCGATTCTGGTGAAACTGCTGAGGCTACCAGACTCAAGAGAACCGCTAGAAGAAGGTACACCAGA
AGAAAGAACAGGATCTGCTACCTCCAAGAGATCTTCTCTAACGAGATGGCTAAAGTGGATGATTCATTCTTCCACAGGCTC
GAAGAGTCATTCCTCGTGGAAGAAGATAAGAAGCACGAGAGGCACCCTATCTTCGGAAACATCGTTGATGAGGTGGCATA
CCACGAGAAGTACCCTACTATCTACCACCTCAGAAAGAAGCTCGTTGATTCTACTGATAAGGCTGATCTCAGGCTCATCTA
CCTCGCTCTCGCTCACATGATCAAGTTCAGAGGACACTTCCTCATCGAGGGTGATCTCAACCCTGATAACTCTGATGTGG
ATAAGTTGTTCATCCAGCTCGTGCAGACCTACAACCAGCTTTTCGAAGAGAACCCTATCAACGCTTCAGGTGTGGATGCTA
AGGCTATCCTCTCTGCTAGGCTCTCTAAGTCAAGAAGGCTTGAGAACCTCATTGCTCAGCTCCCTGGTGAGAAGAAGAAC
GGACTTTTCGGAAACTTGATCGCTCTCTCTCTCGGACTCACCCCTAACTTCAAGTCTAACTTCGATCTCGCTGAGGATGCA
AAGCTCCAGCTCTCAAAGGATACCTACGATGATGATCTCGATAACCTCCTCGCTCAGATCGGAGATCAGTACGCTGATTTG
TTCCTCGCTGCTAAGAACCTCTCTGATGCTATCCTCCTCAGTGATATCCTCAGAGTGAACACCGAGATCACCAAGGCTCCA
CTCTCAGCTTCTATGATCAAGAGATACGATGAGCACCACCAGGATCTCACACTTCTCAAGGCTCTTGTTAGACAGCAGCTC
CCAGAGAAGTACAAAGAGATTTTCTTCGATCAGTCTAAGAACGGATACGCTGGTTACATCGATGGTGGTGCATCTCAAGAA
GAGTTCTACAAGTTCATCAAGCCTATCCTCGAGAAGATGGATGGAACCGAGGAACTCCTCGTGAAGCTCAATAGAGAGGA
TCTTCTCAGAAAGCAGAGGACCTTCGATAACGGATCTATCCCTCATCAGATCCACCTCGGAGAGTTGCACGCTATCCTTAG
AAGGCAAGAGGATTTCTACCCATTCCTCAAGGATAACAGGGAAAAGATTGAGAAGATTCTCACCTTCAGAATCCCTTACTA
CGTGGGACCTCTCGCTAGAGGAAACTCAAGATTCGCTTGGATGACCAGAAAGTCTGAGGAAACCATCACCCCTTGGAAC
TTCGAAGAGGTGGTGGATAAGGGTGCTAGTGCTCAGTCTTTCATCGAGAGGATGACCAACTTCGATAAGAACCTTCCAAA
CGAGAAGGTGCTCCCTAAGCACTCTTTGCTCTACGAGTACTTCACCGTGTACAACGAGTTGACCAAGGTTAAGTACGTGA
CCGAGGGAATGAGGAAGCCTGCTTTTTTGTCAGGTGAGCAAAAGAAGGCTATCGTTGATCTCTTGTTCAAGACCAACAGA
AAGGTGACCGTGAAGCAGCTCAAAGAGGATTACTTCAAGAAAATCGAGTGCTTCGATTCAGTTGAGATTTCTGGTGTTGA
GGATAGGTTCAACGCATCTCTCGGAACCTACCACGATCTCCTCAAGATCATTAAGGATAAGGATTTCTTGGATAACGAGGA
AAACGAGGATATCTTGGAGGATATCGTTCTTACCCTCACCCTCTTTGAAGATAGAGAGATGATTGAAGAAAGGCTCAAGAC
CTACGCTCATCTCTTCGATGATAAGGTGATGAAGCAGTTGAAGAGAAGAAGATACACTGGTTGGGGAAGGCTCTCAAGAA
AGCTCATTAACGGAATCAGGGATAAGCAGTCTGGAAAGACAATCCTTGATTTCCTCAAGTCTGATGGATTCGCTAACAGAA
ACTTCATGCAGCTCATCCACGATGATTCTCTCACCTTTAAAGAGGATATCCAGAAGGCTCAGGTTTCAGGACAGGGTGATA
GTCTCCATGAGCATATCGCTAACCTCGCTGGATCTCCTGCAATCAAGAAGGGAATCCTCCAGACTGTGAAGGTTGTGGAT
GAGTTGGTGAAGGTGATGGGAAGGCATAAGCCTGAGAACATCGTGATCGAAATGGCTAGAGAGAACCAGACCACTCAGA
AGGGACAGAAGAACTCTAGGGAAAGGATGAAGAGGATCGAGGAAGGTATCAAAGAGCTTGGATCTCAGATCCTCAAAGA
GCACCCTGTTGAGAACACTCAGCTCCAGAATGAGAAGCTCTACCTCTACTACCTCCAGAACGGAAGGGATATGTATGTGG
ATCAAGAGTTGGATATCAACAGGCTCTCTGATTACGATGTTGATGCCATCGTGCCACAGTCATTCTTGAAGGATGATTCTAT
CGATAACAAGGTGCTCACCAGGTCTGATAAGAACAGGGGTAAGAGTGATAACGTGCCAAGTGAAGAGGTTGTGAAGAAAA
TGAAGAACTATTGGAGGCAGCTCCTCAACGCTAAGCTCATCACTCAGAGAAAGTTCGATAACTTGACTAAGGCTGAGAGG
GGAGGACTCTCTGAATTGGATAAGGCAGGATTCATCAAGAGGCAGCTTGTGGAAACCAGGCAGATCACTAAGCACGTTG
CACAGATCCTCGATTCTAGGATGAACACCAAGTACGATGAGAACGATAAGTTGATCAGGGAAGTGAAGGTTATCACCCTCA
AGTCAAAGCTCGTGTCTGATTTCAGAAAGGATTTCCAATTCTACAAGGTGAGGGAAATCAACAACTACCACCACGCTCAC
GATGCTTACCTTAACGCTGTTGTTGGAACCGCTCTCATCAAGAAGTATCCTAAGCTCGAGTCAGAGTTCGTGTACGGTGAT
TACAAGGTGTACGATGTGAGGAAGATGATCGCTAAGTCTGAGCAAGAGATCGGAAAGGCTACCGCTAAGTATTTCTTCTAC
TCTAACATCATGAATTTCTTCAAGACCGAGATTACCCTCGCTAACGGTGAGATCAGAAAGAGGCCACTCATCGAGACAAAC
GGTGAAACAGGTGAGATCGTGTGGGATAAGGGAAGGGATTTCGCTACCGTTAGAAAGGTGCTCTCTATGCCACAGGTGA

ACATCGTTAAGAAAACCGAGGTGCAGACCGGTGGATTCTCTAAAGAGTCTATCCTCCCTAAGAGGAACTCTGATAAGCTCA
TTGCTAGGAAGAAGGATTGGGACCCTAAGAAATACGGTGGTTTCGATTCTCCTACCGTGGCTTACTCTGTTCTCGTTGTG
GCTAAGGTTGAGAAGGGAAAGAGTAAGAAGCTCAAGTCTGTTAAGGAACTTCTCGGAATCACTATCATGGAAAGGTCATC
TTTCGAGAAGAACCCAATCGATTTCCTCGAGGCTAAGGGATACAAAGAGGTTAAGAAGGATCTCATCATCAAGCTCCCAAA
GTACTCACTCTTCGAACTCGAGAACGGTAGAAAGAGGATGCTCGCTTCTGCTGGTGAGCTTCAAAAGGGAAACGAGCTT
GCTCTCCCATCTAAGTACGTTAACTTTCTTTACCTCGCTTCTCACTACGAGAAGTTGAAGGGATCTCCAGAAGATAACGAG
CAGAAGCAACTTTTCGTTGAGCAGCACAAGCACTACTTGGATGAGATCATCGAGCAGATCTCTGAGTTCTCTAAAAGGGT
GATCCTCGCTGATGCAAACCTCGATAAGGTGTTGTCTGCTTACAACAAGCACAGAGATAAGCCTATCAGGGAACAGGCAG
AGAACATCATCCATCTCTTCACCCTTACCAACCTCGGTGCTCCTGCTGCTTTCAAGTACTTCGATACAACCATCGATAGGA
AGAGATACACCTCTACCAAAGAAGTGCTCGATGCTACCCTCATCCATCAGTCTATCACTGGACTCTACGAGACTAGGATCG
ATCTCTCACAGCTCGGTGGTGATTCTGGTGGTTCTTCTGGTGGTAGTTCTGGTTCTGAAACTCCTGGTACTTCTGAATCTG
CTACTCCTGAATCTTCTGGTGGATCTTCTGGTGGATCATCTACCCTAAATATAGAAGATGAGTATCGGCTACATGAGACCTC
AAAAGAGCCAGATGTTTCTCTAGGGTCCACATGGCTGTCTGATTTTCCTCAGGCCTGGGCGGAAACCGGGGGCATGGGA
CTGGCAGTTCGCCAAGCTCCTCTGATCATACCTCTGAAAGCAACCTCTACCCCCGTGTCCATAAAACAATACCCCATGTCA
CAAGAAGCCAGACTGGGGATCAAGCCCCACATACAGAGACTGTTGGACCAGGGAATACTGGTACCCTGCCAGTCCCCCT
GGAACACGCCCCTGCTACCCGTTAAGAAACCAGGGACTAATGATTATAGGCCTGTCCAGGATCTGAGAGAAGTCAACAAG
CGGGTGGAAGACATCCACCCCACCGTGCCCAACCCTTACAACCTCTTGAGCGGGCTCCCACCGTCCCACCAGTGGTAC
ACTGTGCTTGATTTAAAGGATGCCTTTTTCTGCCTGAGACTCCACCCCACCAGTCAGCCTCTCTTCGCCTTTGAGTGGAG
AGATCCAGAGATGGGAATCTCAGGACAATTGACCTGGACCAGACTCCCACAGGGTTTCAAAAACAGTCCCACCCTGTTTA
ATGAGGCACTGCACAGAGACCTAGCAGACTTCCGGATCCAGCACCCAGACTTGATCCTGCTACAGTACGTGGATGACTTA
CTGCTGGCCGCCACTTCTGAGCTAGACTGCCAACAAGGTACTCGGGCCCTGTTACAAACCCTAGGGAACCTCGGGTATC
GGGCCTCGGCCAAGAAAGCCCAAATTTGCCAGAAACAGGTCAAGTATCTGGGGTATCTTCTAAAAGAGGGTCAGAGATG
GCTGACTGAGGCCAGAAAAGAGACTGTGATGGGGCAGCCTACTCCGAAGACCCCTCGACAACTAAGGGAGTTCCTAGG
GAAGGCAGGCTTCTGTCGCCTCTTCATCCCTGGGTTTGCAGAAATGGCAGCCCCCCTGTACCCTCTCACCAAACCGGGG
ACTCTGTTTAATTGGGGCCCAGACCAACAAAAGGCCTATCAAGAAATCAAGCAAGCTCTTCTAACTGCCCCAGCCCTGGG
GTTGCCAGATTTGACTAAGCCCTTTGAACTCTTTGTCGACGAGAAGCAGGGCTACGCCAAAGGTGTCCTAACGCAAAAAC
TGGGACCTTGGCGTCGGCCGGTGGCCTACCTGTCCAAAAAGCTAGACCCAGTAGCAGCTGGGTGGCCCCCTTGCCTAC
GGATGGTAGCAGCCATTGCCGTACTGACAAAGGATGCAGGCAAGCTAACCATGGGACAGCCACTAGTCATTCTGGCCCC
CCATGCAGTAGAGGCACTAGTCAAACAACCCCCCGACCGCTGGCTTTCCAACGCCCGGATGACTCACTATCAGGCCTTG
CTTTTGGACACGGACCGGGTCCAGTTCGGACCGGTGGTAGCCCTGAACCCGGCTACGCTGCTCCCACTGCCTGAGGAA
GGGCTGCAACACAACTGCCTTGATATCCTGGCCGAAGCCCACGGAACCCGACCCGACCTAACGGACCAGCCGCTCCCA
GACGCCGACCACACCTGGTACACGGATGGAAGCAGTCTCTTACAAGAGGGACAGCGTAAGGCGGGAGCTGCGGTGACC
ACCGAGACCGAGGTAATCTGGGCTAAAGCCCTGCCAGCCGGGACATCCGCTCAGCGGGCTGAACTGATAGCACTCACC
CAGGCCCTAAAGATGGCAGAAGGTAAGAAGCTAAATGTTTATACTGATAGCCGTTATGCTTTTGCTACTGCCCATATCCATG
GAGAAATATACAGAAGGCGTGGGTGGCTCACATCAGAAGGCAAAGAGATCAAAAATAAAGACGAGATCTTGGCCCTACTA
AAAGCCCTCTTTCTGCCCAAAAGACTTAGCATAATCCATTGTCCAGGACATCAAAAGGGACACAGCGCCGAGGCTAGAGG
CAACCGGATGGCTGACCAAGCGGCCCGAAAGGCAGCCATCACAGAGACTCCAGACACCTCTACCCTCCTCATAGAAAAT
TCATCACCCTCTGGCGGCTCAAAAAGAACCGCCGACGGCAGCGAATTCGAGCCAAAGAAAAAGAGAAAGTAGGCTCC
GGAGAGGGCAGAGGATCCCTGCTAACATGCGGTGATGTCGAGGAGAATCCTGGCCCAATGGTGAGCAAGGGCGAGGAG
CTGTTCACCGGGGTGGTGCCCATCCTGGTCGAGCTGGACGGCGACGTAAACGGCCACAAGTTCAGCGTGTCCGGCGA
GGGCGAGGGCGATGCCACCTACGGCAAGCTGACCCTGAAGTTCATCTGCACCACCGGCAAGCTGCCCGTGCCCTGGC
CCACCCTCGTGACCACCTTCACCTACGGCGTGCAGTGCTTCAGCCGCTACCCCGACCACATGAAGCAGCACGACTTCTT
CAAGTCCGCCATGCCCGAAGGCTACGTCCAGGAGCGCACCATCTTCTTCAAGGACGACGGCAACTACAAGACCCGCGC
CGAGGTGAAGTTCGAGGGCGACACCCTGGTGAACCGCATCGAGCTGAAGGGCATCGACTTCAAGGAGGACGGCAACAT
CCTGGGGCACAAGCTGGAGTACAACTACAACAGCCACAACGTCTATATCATGGCCGACAAGCAGAAGAACGGCATCAAG
GTGAACTTCAAGATCCGCCACAACATCGAGGACGGCAGCGTGCAGCTCGCCGACCACTACCAGCAGAACACCCCCATC
GGCGACGGCCCCGTGCTGCTGCCCGACAACCACTACCTGAGCACCCAGTCCGCCCTGAGCAAAGACCCCAACGAGAA
GCGCGATCACATGGTCCTGCTGGAGTTCGTGACCGCCGCCGGGATCACTCACGGCATGGACGAGCTGTACAAGTAA

Arabidopsis codon-optimized nCas9(H840A)
Arabidopsis codon-optimized RT linker
MLV reverse transcriptase
T2A linker
EGFP protein
NLS

pS8K-nCas9RT:

ATGGATAAGAAATACTCAATAGGCTTAGATATCGGCACAAATAGCGTCGGATGGGCGGTGATCACTGATGAATATAAGGTTC
CGTCTAAAAAGTTCAAGGTTCTGGGAAATACAGACCGCCACAGTATCAAAAAAAATCTTATAGGGGCTCTTTTATTTGACAG
TGGAGAGACAGCGGAAGCGACTCGCCTCAAACGGACAGCTCGTAGAAGGTATACACGTCGGAAGAATCGTATTTGTTATC
TACAGGAGATTTTTTCAAATGAGATGGCGAAAGTAGATGATAGTTTCTTTCATCGACTTGAAGAGTCTTTTTTGGTGGAAGA
AGACAAGAAGCATGAACGTCATCCTATTTTTGGAAATATAGTAGATGAAGTTGCTTATCATGAGAAATATCCAACTATCTATCA
TCTGCGAAAAAAATTGGTAGATTCTACTGATAAAGCGGATTTGCGCTTAATCTATTTGGCCTTAGCGCATATGATTAAGTTTC
GTGGTCATTTTTTGATTGAGGGAGATTTAAATCCTGATAATAGTGATGTGGACAAACTATTTATCCAGTTGGTACAAACCTAC
AATCAATTATTTGAAGAAAACCCTATTAACGCAAGTGGAGTAGATGCTAAAGCGATTCTTTCTGCACGATTGAGTAAATCAA
GACGATTAGAAAATCTCATTGCTCAGCTCCCCGGTGAGAAGAAAAATGGCTTATTTGGGAATCTCATTGCTTTGTCATTGG
GTTTGACCCCTAATTTTAAATCAAATTTTGATTTGGCAGAAGATGCTAAATTACAGCTTTCAAAAGATACTTACGATGATGATT
TAGATAATTTATTGGCGCAAATTGGAGATCAATATGCTGATTTGTTTTTGGCAGCTAAGAATTTATCAGATGCTATTTTACTTT

CAGATATCCTAAGAGTAAATACTGAAATAACTAAGGCTCCCCTATCAGCTTCAATGATTAAACGCTACGATGAACATCATCAA
GACTTGACTCTTTTAAAAGCTTTAGTTCGACAACAACTTCCAGAAAAGTATAAAGAAATCTTTTTTGATCAATCAAAAAACGG
ATATGCAGGTTATATTGATGGGGGAGCTAGCCAAGAAGAATTTTATAAATTTATCAAACCAATTTTAGAAAAAATGGATGGTA
CTGAGGAATTATTGGTGAAACTAAATCGTGAAGATTTGCTGCGCAAGCAACGGACCTTTGACAACGGCTCTATTCCCCATC
AAATTCACTTGGGTGAGCTGCATGCTATTTTGAGAAGACAAGAAGACTTTTATCCATTTTTAAAAGACAATCGTGAGAAGAT
TGAAAAAATCTTGACTTTTCGAATTCCTTATTATGTTGGTCCATTGGCGCGTGGCAATAGTCGTTTTGCATGGATGACTCGG
AAGTCTGAAGAAACAATTACCCCATGGAATTTTGAAGAAGTTGTCGATAAAGGTGCTTCAGCTCAATCATTTATTGAACGCA
TGACAAACTTTGATAAAAATCTTCCAAATGAAAAAGTACTACCAAAACATAGTTTGCTTTATGAGTATTTTACGGTTTATAACG
AATTGACAAAGGTCAAATATGTTACTGAAGGAATGCGAAAACCAGCATTTCTTTCAGGTGAACAGAAGAAAGCCATTGTTG
ATTTACTCTTCAAAACAAATCGAAAAGTAACCGTTAAGCAATTAAAAGAAGATTATTTCAAAAAAATAGAATGTTTTGATAGTG
TTGAAATTTCAGGAGTTGAAGATAGATTTAATGCTTCATTAGGTACCTACCATGATTTGCTAAAAATTATTAAAGATAAAGATT
TTTTGGATAATGAAGAAAATGAAGATATCTTAGAGGATATTGTTTTAACATTGACCTTATTTGAAGATAGGGAGATGATTGAG
GAAAGACTTAAAACATATGCTCACCTCTTTGATGATAAGGTGATGAAACAGCTTAAACGTCGCCGTTATACTGGTTGGGGA
CGTTTGTCTCGAAAATTGATTAATGGTATTAGGGATAAGCAATCTGGCAAAACAATATTAGATTTTTTGAAATCAGATGGTTTT
GCCAATCGCAATTTTATGCAGCTGATCCATGATGATAGTTTGACATTTAAAGAAGACATTCAAAAAGCACAAGTGTCTGGAC
AAGGCGATAGTTTACATGAACATATTGCAAATTTAGCTGGTAGCCCTGCTATTAAAAAAGGTATTTTACAGACTGTAAAAGTT
GTTGATGAATTGGTCAAAGTAATGGGGCGGCATAAGCCAGAAAATATCGTTATTGAAATGGCACGTGAAAATCAGACAACT
CAAAAGGGCCAGAAAAATTCGCGAGAGCGTATGAAACGAATCGAAGAAGGTATCAAAGAATTAGGAAGTCAGATTCTTAAA
GAGCATCCTGTTGAAAATACTCAATTGCAAAATGAAAAGCTCTATCTCTATTATCTCCAAAATGGAAGAGACATGTATGTGG
ACCAAGAATTAGATATTAATCGTTTAAGTGATTATGATGTCGATGCGATTGTTCCACAAAGTTTCCTTAAAGACGATTCAATA
GACAATAAGGTCTTAACGCGTTCTGATAAAAATCGTGGTAAATCGGATAACGTTCCAAGTGAAGAAGTAGTCAAAAAGATG
AAAAACTATTGGAGACAACTTCTAAACGCCAAGTTAATCACTCAACGTAAGTTTGATAATTTAACGAAAGCTGAACGTGGAG
GTTTGAGTGAACTTGATAAAGCTGGTTTTATCAAACGCCAATTGGTTGAAACTCGCCAAATCACTAAGCATGTGGCACAAA
TTTTGGATAGTCGCATGAATACTAAATACGATGAAAATGATAAACTTATTCGAGAGGTTAAAGTGATTACCTTAAAATCTAAAT
TAGTTTCTGACTTCCGAAAAGATTTCCAATTCTATAAAGTACGTGAGATTAACAATTACCATCATGCCCATGATGCGTATCTA
AATGCCGTCGTTGGAACTGCTTTGATTAAGAAATATCCAAAACTTGAATCGGAGTTTGTCTATGGTGATTATAAAGTTTATGA
TGTTCGTAAAATGATTGCTAAGTCTGAGCAAGAAATAGGCAAAGCAACCGCAAAATATTTCTTTTACTCTAATATCATGAACT
TCTTCAAAACAGAAATTACACTTGCAAATGGAGAGATTCGCAAACGCCCTCTAATCGAAACTAATGGGGAAACTGGAGAAA
TTGTCTGGGATAAAGGGCGAGATTTTGCCACAGTGCGCAAAGTATTGTCCATGCCCCAAGTCAATATTGTCAAGAAAACAG
AAGTACAGACAGGCGGATTCTCCAAGGAGTCAATTTTACCAAAAAGAAATTCGGACAAGCTTATTGCTCGTAAAAAAGACT
GGGATCCAAAAAAATATGGTGGTTTTGATAGTCCAACGGTAGCTTATTCAGTCCTAGTGGTTGCTAAGGTGGAAAAAGGGA
AATCGAAGAAGTTAAAATCCGTTAAAGAGTTACTAGGGATCACAATTATGGAAAGAAGTTCCTTTGAAAAAAATCCGATTGA
CTTTTTAGAAGCTAAAGGATATAAGGAAGTTAAAAAAGACTTAATCATTAAACTACCTAAATATAGTCTTTTTGAGTTAGAAAA
CGGTCGTAAACGGATGCTGGCTAGTGCCGGAGAATTACAAAAAGGAAATGAGCTGGCTCTGCCAAGCAAATATGTGAATT
TTTTATATTTAGCTAGTCATTATGAAAAGTTGAAGGGTAGTCCAGAAGATAACGAACAAAAACAATTGTTTGTGGAGCAGCAT
AAGCATTATTTAGATGAGATTATTGAGCAAATCAGTGAATTTTCTAAGCGTGTTATTTTAGCAGATGCCAATTTAGATAAAGTT
CTTAGTGCATATAACAAACATAGAGACAAACCAATACGTGAACAAGCAGAAAATATTATTCATTTATTTACGTTGACGAATCTT
GGAGCTCCCGCTGCTTTTAAATATTTTGATACAACAATTGATCGTAAACGATATACGTCTACAAAAGAAGTTTTAGATGCCAC
TCTTATCCATCAATCCATCACTGGTCTTTATGAAACACGCATTGATTTGAGTCAGCTAGGAGGTGACAGTGGCGGTAGTAG
CGGCGGTAGTAGTGGCAGTGAAACCCCGGGTACCAGTGAAAGCGCGACCCCAGAAAGCAGTGGTGGCAGTAGCGGTG
GCAGCAGCACCCTCAATATCGAAGACGAGTACCGTCTGCACGAAACCAGCAAAGAGCCGGATGTGAGTCTGGGCAGCA
CGTGGCTCAGTGATTTCCCACAAGCGTGGGCCGAAACGGGTGGTATGGGTCTGGCGGTGCGTCAAGCCCCACTGATTAT
TCCACTGAAGGCGACGAGCACCCCAGTGAGCATCAAGCAGTACCCAATGAGTCAAGAAGCGCGTCTGGGCATCAAACC
GCATATTCAACGTCTGCTGGATCAAGGCATCCTCGTTCCATGCCAGAGCCCGTGGAATACGCCGCTGCTGCCGGTGAAA
AAACCGGGTACGAACGACTACCGCCCAGTGCAAGATCTGCGCGAAGTGAACAAACGCGTGGAAGACATCCACCCGACG
GTGCCAAACCCATACAATCTGCTGAGTGGTCTGCCACCGAGTCACCAGTGGTATACGGTTCTGGATCTGAAAGACGCGTT
CTTCTGTCTGCGCCTCCATCCAACGAGCCAGCCGCTCTTCGCCTTTGAATGGCGCGATCCGGAAATGGGCATCAGTGGT
CAACTGACGTGGACCCGTCTGCCGCAAGGCTTCAAGAACAGCCCGACGCTCTTCAATGAAGCGCTGCACCGCGATCTG
GCGGACTTTCGTATCCAGCACCCAGATCTGATTCTGCTCCAGTACGTGGACGATCTGCTGCTGGCGGCGACCAGCGAAC
TGGATTGCCAGCAAGGTACGCGTGCGCTGCTGCAAACCCTCGGCAATCTCGGCTACCGTGCCAGCGCCAAAAAGGCCC
AGATCTGCCAAAAGCAAGTTAAGTACCTCGGCTATCTGCTCAAGGAGGGCCAGCGTTGGCTGACGGAGGCCCGTAAAGA
AACCGTTATGGGCCAGCCGACGCCGAAAACGCCACGTCAGCTGCGCGAATTTCTGGGTAAAGCCGGCTTTTGTCGTCTG
TTCATCCCGGGCTTCGCGGAGATGGCGGCGCCACTGTACCCACTCACGAAACCGGGCACCCTCTTCAACTGGGGTCCG
GATCAGCAGAAGGCCTACCAAGAAATCAAACAAGCGCTGCTGACGGCGCCAGCGCTGGGCCTCCCAGATCTGACCAAA
CCATTCGAGCTCTTCGTTGACGAGAAGCAAGGCTACGCCAAAGGCGTTCTCACGCAGAAACTGGGTCCATGGCGTCGC
CCAGTTGCGTATCTGAGCAAAAAGCTGGATCCAGTTGCCGCGGGTTGGCCACCATGTCTGCGCATGGTTGCGGCGATTG
CGGTTCTGACGAAAGATGCCGGCAAACTGACCATGGGTCAACCGCTCGTGATTCTCGCCCCGCATGCGGTGGAAGCGC
TGGTGAAACAACCACCGGATCGTTGGCTGAGCAATGCCCGTATGACGCATTACCAAGCGCTGCTGCTCGATACCGATCG
CGTGCAGTTTGGCCCAGTGGTTGCCCTCAATCCGGCGACGCTGCTGCCACTGCCGGAAGAAGGCCTCCAGCACAACTG
TCTGGATATTCTGGCCGAAGCCCATGGTACGCGTCCGGATCTCACCGATCAGCCGCTGCCAGATGCGGACCATACGTGG
TACACCGACGGCAGTAGTCTGCTGCAAGAAGGTCAACGTAAAGCGGGTGCCGCGGTGACCACGGAAACGGAAGTGATC
TGGGCCAAAGCGCTCCCAGCCGGTACGAGTGCCCAACGTGCCGAACTGATCGCGCTGACCCAAGCGCTGAAGATGGC
GGAGGGCAAAAAGCTCAACGTGTACACGGACAGCCGTTATGCCTTTGCCACGGCGCACATTCATGGTGAAATCTATCGC
CGTCGCGGCTGGCTGACCAGCGAAGGCAAGGAGATCAAGAACAAGGACGAGATTCTGGCGCTCCTCAAAGCCCTCTTT
CTGCCGAAACGTCTGAGCATTATCCACTGCCCGGGCCATCAGAAAGGTCACAGTGCCGAGGCCCGTGGCAACCGTATG
GCGGATCAAGCCGCGCGTAAGGCGGCCATCACCGAAACGCCAGATACGAGTACGCTGCTGATCGAAAATAGCAGCCCAT
GA

E.coli codon-optimized nCas9(H840A)

E.coli codon-optimized RT linker
E.coli codon-optimized MLV reverse transcriptase

2. Sequences of promotors in protoplast used in this study

2×35S promotor:

GGGCCCCCCCTCGAGGTCGACGGTATCGATAAGCTTGCATGCCTGCAGGTCAACATGGTGGAGCACGACACACTTGTCT
ACTCCAAAAATATCAAAGATACAGTCTCAGAAGACCAAAGGGCAATTGAGACTTTTCAACAAAGGGTGATATCCGGAAACC
TCCTCGGATTCCATTGCCCAGCTATCTGTCACTTTATTGTGAAGATAGTGGAAAAGGAAGGTGGCTCCTACAAATGCCATC
ATTGCGATAAAGGAAAGGCCATCGTTGAAGATGCCTCTGCCGACAGTGGTCCCAAAGATGGACCCCCACCCACGAGGAG
CATCGTGGAAAAAGAAGACGTTCCAACCACGTCTTCAAAGCAAGTGGATTGATGTGATAACATGGTGGAGCACGACACAC
TTGTCTACTCCAAAAATATCAAAGATACAGTCTCAGAAGACCAAAGGGCAATTGAGACTTTTCAACAAAGGGTAATATCCGG
AAACCTCCTCGGATTCCATTGCCCAGCTATCTGTCACTTTATTGTGAAGATAGTGGAAAAGGAAGGTGGCTCCTACAAATG
CCATCATTGCGATAAAGGAAAGGCCATCGTTGAAGATGCCTCTGCCGACAGTGGTCCCAAAGATGGACCCCCACCCACG
AGGAGCATCGTGGAAAAAGAAGACGTTCCAACCACGTCTTCAAAGCAAGTGGATTGATGTGATATCTCCACTGACGTAAG
GGATGACGCACAATCCCACTATCCTTCGCAAGACCCTTCCTCTATATAAGGAAGTTCATTTCATTTGGAGAGGACCTCGAC
CTCAACACAACATATACAAAACAAACGAATCTCAAGCAATCAAGCATTCTACTTCTATTGCAGCAATTTAAATCATTTCTTTTA
AAGCAAAAGCAATTTTCTGAAAATTTTCACCATTTACGAACGATA

RPS5A promotor:

CTCAACTTTTGATTCGCTATTTGCAGTGCACCTGTGGCGTTCATCACATCTTTTGTGACACTGTTTGCACTGGTCATTGCTA
TTACAAAGGACCTTCCTGATGTTGAAGGAGATCGAAAGTAAGTAACTGCACGCATAACCATTTTCTTTCCGCTCTTTGGCT
CAATCCATTTGACAGTCAAAGACAATGTTTAACCAGCTCCGTTTGATATATTGTCTTTATGTGTTTGTTCAAGCATGTTTAGT
TAATCATGCCTTTGATTGATCTTGAATAGGTTCCAAATATCAACCCTGGCAACAAAACTTGGAGTGAGAAACATTGCATTCC
TCGGTTCTGGACTTCTGCTAGTAAATTATGTTTCAGCCATATCACTAGCTTTCTACATGCCTCAGGTGAATTCATCTATTTCC
GTCTTAACTATTTCGGTTAATTAAAGCACGAACACCATTACTGCATGTAGAAGCTTGATAAACTATCGCCACCAATTTATTTTT
GTTGCGATATTGTTACTTTCCTCAGTATGCAGCTTTGAAAAGACCAACCCTCTTATCCTTTAACAATGAACAGGTTTTTAGA
GGTAGCTTGATGATTCCTGCACATGTGATCTTGGCTTCAGGCTTAATTTTCCAGGTAAAGCATTATGAGATACTCTTATATCT
CTTACATACTTTTGAGATAATGCACAAGAACTTCATAACTATATGCTTTAGTTTCTGCATTTGACACTGCCAAATTCATTAATC
TCTAATATCTTTGTTGTTGATCTTTGGTAGACATGGGTACTAGAAAAAGCAAACTACACCAAGGTAAAATACTTTTGTACAAA
CATAAACTCGTTATCACGGAACATCAATGGAGTGTATATCTAACGGAGTGTAGAAACATTTGATTATTGCAGGAAGCTATCTC
AGGATATTATCGGTTTATATGGAATCTCTTCTACGCAGAGTATCTGTTATTCCCCTTCCTCTAGCTTTCAATTTCATGGTGAG
GATATGCAGTTTTCTTTGTATATCATTCTTCTTCTTCTTTGTAGCTTGGAGTCAAAATCGGTTCCTTCATGTACATACATCAAG
GATATGTCCTTCTGAATTTTTATATCTTGCAATAAAAATGCTTGTACCAATTGAAACACCAGCTTTTTGAGTTCTATGATCACT
GACTTGGTTCTAACCAAAAAAAAAAAAATGTTTAATTTACATATCTAAAAGTAGGTTTAGGGAAACCTAAACAGTAAAATATTTG
TATATTATTCGAATTTCACTCATCATAAAAACTTAAATTGCACCATAAAATTTTGTTTTACTATTAATGATGTAATTTGTGTAACT
TAAGATAAAAATAATATTCCGTAAGTTAACCGGCTAAAACCACGTATAAACCAGGGAACCTGTTAAACCGGTTCTTTACTGGA
TAAAGAAATGAAAGCCCATGTAGACAGCTCCATTAGAGCCCAAACCCTAAATTTCTCATCTATATAAAAGGAGTGACATTAG
GGTTTTTGTTCGTCCTCTTAAAGCTTCTCGTTTTCTCTGCCGTCTCTCTCATTCGCGCGACGCAAACGATCTTCAGGTGAT
CTTCTTTCTCCAAATCCTCTCTCATAACTCTGATTTCGTACTTGTGTATTTGAGCTCACGCTCTGTTTCTCTCACCACAGCC

UBQ10 promotor:

GTCGACGAGTCAGTAATAAACGGCGTCAAAGTGGTTGCAGCCGGCACACACGAGTCGTGTTTATCAACTCAAAGCACAAA
TACTTTTCCTCAACCTAAAAATAAGGCAATTAGCCAAAAACAACTTTGCGTGTAAACAACGCTCAATACACGTGTCATTTTAT
TATTAGCTATTGCTTCACCGCCTTAGCTTTCTCGTGACCTAGTCGTCCTCGTCTTTTCTTCTTCTTCTTCTATAAAACAATAC
CCAAAGAGCTCTTCTTCTTCACAATTCAGATTTCAATTTCTCAAAATCTTAAAAACTTTCTCTCAATTCTCTCTACCGTGATC
AAGGTAAATTTCTGTGTTCCTTATTCTCTCAAAATCTTCGATTTTGTTTTCGTTCGATCCCAATTTCGTATATGTTCTTTGGTT
TAGATTCTGTTAATCTTAGATCGAAGACGATTTTCTGGGTTTGATCGTTAGATATCATCTTAATTCTCGATTAGGGTTTCATAG
ATATCATCCGATTTGTTCAAATAATTTGAGTTTTGTCGAATAATTACTCTTCGATTTGTGATTTCTATCTAGATCTGGTGTTAGT
TTCTAGTTTGTGCGATCGAATTTGTCGATTAATCTGAGTTTTTCTGATTAACAG

3. Sequences of other reverse transcriptase(RT) used in this study

Fs-RT:

TTTACCATCGACGAGATGCTGAGCAAGAACAACCAACGTCTGGCGTTCGAACACTTTGCGACCAAAAACGACGGTTGCG
GCCCGGACGGTATGCACGTTAGCGAACTGGAGAAGTACTGGCGTATGAATCACGACCAGATCATTAGCGATCTGAAGAAC
CAGGAATACCAGCCGGGTATTATCCTGATTCGTGAGCACATGAATAAAACCGGCAAACGCCGTAATATCGCGAGCCTGAA
CGTTATTGATCGTTTCATTACCCGTCTGCTGAGCCAGAAACTGAACCGTTATCTGGCGCCGATTTTCTGCGAGAACAGCTA
TGCGTACCAGGACAGCAAAGGCGTTATGCCGGCGGTTCTGAAAGCGAAGGAGTACGTTGAGCTGGGTATGCGTCACGT
GATTGAAATCGATCTGAAAAACTACTTTGACACCATTCCGCTGGAAAACCTGATCCCGGAGATCGAACGCTACATCACCGA
TGAGGCGGTTCTGCATCTGATCAAGCAATACCTGTTTTGCGATATTAGCTTCGAGGGCAAAATCAGCCGTAAAACCCAAGG
TATCGTTCAAGGTAACGCGATCAGCCCGATCCTGAGCAACCTGTACCTGAACGACTTCGACAAGGAACTGGACGAGAGC
AAGCTGTGCTGGATTCGTTACGCGGACAATATCTACATCTATATGGATAGCTATGAGAAGGCGCTGCTGGTGTATAGCGAG

CTGACCGAGCGTCTGGAGCGTCGCAAGCTGACCGTTAACAAGGAGAAAAGCGGCGTTTTTGATGTGAGCACCCGTAGCA
TTCTGGGCTACGACATCCTGATTCGTAACAAGAAAGTGGACGTTCGTAAGCATATTTACAAAAGCGTTAATCAGTACAGCA
ACTGGCACGATAGCCGCCTGGAGTTTATCAACGGTCGTTACCACATCACCAGCGATGGCATCCTGAATCGCCAAGACTTC
GGCCTGCTGTTCGAGAATGAGCAGAAAAAACACTACATCCCGGTTGAGGTTAGCGATCAGCTGAACATCTACGGTAATGT
TACCCTGGCGAGCAATGTGCTGCAAAGTTTCAGCAACCGCGAAATCAAAGTGAGCTTCTTCGACAAGTATGGTCGTCTGA
TTGGTAGCTTCCTGCCGGAAAAAACCAAGAAAAGCGCGGAGATCATTCTGGTGCAGAGCAAGAACTATCTGAACGAAGAT
GTGCGTATGGATACCGCGCGCCGTATGGAAATTGCGGGTCTGCACAACATCCGTGCGAATCTGCGCTACTACGATAAGAA
ACACAAGGGCGACTTTAAAGAGAAGGTTGACGCGATTAGCGGCTACATTGACGCGCTGAACCGTGCGCCGAGCGTTAAT
GATATGATGCTGCTGGAAGCGAAAGCGCGCCAACTGTATTATACCTGTTTTAACCAAATCCTGGAAACCAGCGACTTCCAG
TTCGAAAAGCGTACCAAGCGTCCGCCGAAAGATGCGATCAATGCGTGCATTAGCTTCGGCAATACCCTGCTGTATAATCTA
TTTGTGAATATTATCTGGAAGAAGGGTCTGGACCCGCGCTTTGGCGTTGTTCACGCGAGCAACAAACGCAATCAAAGCCT
GAACCTGGACTTCGCGGACATCTTTAAACCGATCGTGATTGACCGCATTATCTTCACCATGATCAATAAGAAAATGCTGAC
CCTGCTGACCGATTTTGAAACCAGCAACCAGGGTGTGTATCTGAGCCGTGAGGGCAAGAACATCTTCCTGCAAATGTACG
AGGAGAAGCTGAAAAGCCGCATTACCATCAAGGGTAAAGAGATGAGCTATTACCAGCTGCTGGAGAGCGAAGTGCAGAA
TTACAAGAATTTCATTCTGACCGGCGAAACCTATAAACCGTATAAGTATTAT

Mm-RT:

CTGAATTCCCCTCTTATAGACGCCGTTTTGCCTTTACGCAGTGTGGTCATCACACTGCGTTGGCTGTCGCCAAGCAAAAC
GGGCTTTTTGCATCATGCGGGTTTGCACGCTTGGGTACGCTTTCTCGCTGGCAGTCCAGAACAGTTTAGCGACTTTATCG
TCGTCGAACCTATAGAAAACGGCCACATTAGTTACCAAGCAGGCGACGGTTACAGGTTTCGAATTACCGTGTTAAACGGT
GGTGAAAGCCTGCTCGATACGCTGTTCTCTTCGTTAAAACGGCTCCCAGAAAGCGCCGCTAATCACCCAGACATAGCGG
GCGCTTTCTCGGACAACCTCGTCTTAGAAAAAATCGAAGATACATTTGAGCACCATCAAGTCACACAAATAGAAGACCTAA
GCGTGTTTGATATTAATGCGCTAATGCTCGAAACCGCGGTTTGGTCACGTCAACGACGGTTCAAAGTGGCCTTCAACACC
CCAGCGAGGCTCGTCAAACCCAAGCCAGAAGATGGCACAGAGCTAAAAGGCCAAAACCGTTACTGCCGTGATAAATCCG
ACTTAAACTGGCAGCTCTTTACGCATCGCCTCACCGATACCTTTATTAACCTCTTTCAAAGCCGAACTGGCGAGCGTTTAC
AAAGGCAAAACTGGCCCGAAGCGCAACTCCACGCAGGGTTAGCGGTTTGGCTAAACAACAGTTACACCAATAAAAAAGA
GAAAAAAGTCAAAGACGCCAGCGGCATGTTGGCGCAAATGCAAATCGAGATTGATGACGACTTCCCTGCCGATTTACTCG
CGCTTCTTGTTCTGGGTCAATACATCGGCATGGGCCAAAATCGCGCATTCGGTATGGGGCAATATCAACTGCAAGATGCTT
ATGGTTACTGCAGCTACCCTCGCCCTCAAGCGGCCAAAAGTCTGCTCGAAAAAAGTCTATCTGATGCAAGCTTACACCAA
GCCTGCCAAACCATGTACCCAAGACAAGCTAACTTTGACTCGTCGGACACCGACGAAGAACATCACGACGCCATCGACG
AGCTGCTTACCAAACTCTATGTAAGCCGCGAACGTATTTTCAAACGAGAGTTTACTCCAAGCCAGTTACACTCTGTCGAAA
TCGAAAAGCCAGAAGGCGGAACACGACTGCTGTCGGTTCCCAATTGGCACGACCGCACCTTACAAAAAGCGGTAACAGA
ATGCCTTGGCAACACCCTAGAACACATATGGATGAAGCACAGCTATGGCTACCGAAAGGGACACTCTCGCCTACAGGCCC
GAGATCAAATCAACCAATACATTCAGCAAGGCTACGAATGGGTACTCGAAAGCGACATAGAAAGCTTCTTCGACAGTGTCA
ATTGGCTGAACCTAGAACAACGACTGAAACTACTATTGCCTAATGAACCGTTAGTCCCCTTACTCATGCAATGGGTCAGTG
CAGCGAAACAAACCGAAGATGAGCAAACGCTTGCGCGCCATAACGGCCTGCCACAAGGCGCGCCGATCAGTCCGATAC
TGGCTAACCTATTACTTGATGACCTCGACCAAGATATGATCGCCAAAGGGCATCAAATCGTCCGCTACGCTGATGATTTTGT
CTTACTTTTTAAATCAAAAGCGGCGGCAGAAAGCGCACTCGACGACATAATAACAGCCCTTAAAGAGCATCATCTCGCTAT
CAACCTTGAAAAAACGCGTATTGTCGAAGCGTCGCAAGGGTTCCGCTATTTAGGCTACTTATTTGTCGATGGCTACGCGAT
CGAAACCAAACGCGAATACCGCAAAGAGCACGCTCAACTCGACAAACAGCTGAACGCCTCTTCGCTCGAAAACGAGCCC
TCTCTACAACAGGAACCTGCTGTACAAAACGAACAAAGCACGCTTATTGGAGAGCGCGAAAAGCTAGGCACATTGCTCAT
CATAGCAGGGGACATTGCGATGCTAAGCAGTGAAAAACAACGCCTAATAGTCGAACAATACGACGAGCTGCATACTTACC
CTTGGGCCACGTTGAGTTCCGTGTTGCTTGTAGGCCCGCACCACATCACCACCCCCGCTCTAAAATCGGCTATGTTCCAC
AATGTACCCGTACACTTTGCTAGCCAATATGGCCGCTACCAAGGGGTTTCCGCAGGTGCTGCGCCCAGTGTGTTTGGCG
CCGATTTTTGGTTACTTCAAGCTCAATACTTACAACAAGAAACAAACGCACTCAACATTAGCCAAGTGCTAATACAAGCCAG
AATAGAGGGTATAAGAGCCGTCATAAGCCGTAGAGAAAAAGATGCCCCTGAGCTGAACAAAATTCAACGCTTGGACGAAA
AACGCTTACGGGCAGAAACATTAGATCAATTACGAGGCTATGAAGGCCAAGCCAGCAAACAACTGTGGGCATTCTTCCAG
CGTATACTCGAAGAAGATTGGGGCTTTACAGGGCGTAATAGACGCCCTCCCAAAGACCCGATCAACGCCCTACTTTCATTA
GGCTACACCTATCTATACAGCTTAGTAGACAGTGTCAATAGAACGGTTGGACTCTACCCTTGGCAAGGCGCACTGCACCA
GCGTCATGGTTACCATCACACCCTAGCTTCTGACCTAATGGAACCTTGGCGTTACCTTGTCGAACACGTCGTCTTAACCCT
AATAAATCGTCACCAAATACATAAGGATGACTTTGTCATCAAAGAAAATGGTTGCGAAATGAGCAGTGGCGCGAGAAAAAC
ACTTCTAAAAGAACTCTTGGTTCAACTGACTAAAGTGCCTAAGGGAGGAAACAGTCTGCTCACCGAAATGAGCAACCAAT
CTTACCGTTTAGCCCTAAGCTGCAAAATGCAACAACGCTTTATCGCTTGGAGCCCTAAAAGA

Vv-RT:

GCTATTCACCTTACGATTGAGGAATTGCAAACAAGCTGGAAGAAGGTGTCTGCCAATAAAGGCATTGCAGGGGTCGATGG
CGTTACCATTCAAACATTTGCAATCCACTTGGATACCAACTTAAACACGTTACTCAGCGCATGGAACCACGGCAACTATGC
GCCGTCACCCTATCGTCCACTCACCATTCAACCCAACGAAAAGAAAACCCGGCAGCTTGCCATCCCCACCGTTGCTGAC
CGGATTATTCATACCGCCATCGCACAAAAGTTGGTTGCCAAGTTTGAACCAGAGTTTGAACATATAAGCTACGGCTATCGA
CCGAATCGCTCCTACACCCATGCAATTCGTCATATCGAACAACTGCGAAATCAAGGCTACCTATACGTTCTTGATGCGGATA
TCAAAGGTTACTTCGACCATATCTGCCACAAGCGGCTCAAACAGATTTTGCAAAAATACCTTGAGGACAACTGGGTTGAAT
CGATCATGACGCTGCTATTGTCGCAGCAAATGCCCGCGCAAACCTTATTGTTTGGTGTTGAACTGGGGCGCGGCATCCCA
CAAGGTTCGCCACTTTCTCCGCTGCTGGCCAATCTCTATTTGGACGGGTTTGACGAAGCCCTGCTCGATAGAGGTGAGC
AAATCGTTCGATATGCCGACGATTTTGTTGTTCTGGTCACCCACGAACAACAAGCCCAACACTGTTTAGCTTTTGTTACAC
AATACTTAGCGAGTTTAAAACTCCAGTTAAATACTGAGAAAACTCGGGTTGTTAGCTTTCAAGATGGTTTTACTTTTCTTGGT
GTCTCATTT

At-RT:

ACTCTTAATATTGAAGATGAGTATAGGCTTCATGAAACTTCTAAGGAACCTGATGTTTCTTTGGGATCTACTTGGCTTTCAGA
TTTTCCTCAAGCATGGGCTGAAACTGGAGGAATGGGACTTGCTGTGAGACAAGCTCCTCTTATAATTCCTCTTAAAGCTAC
TTCTACTCCTGTTAGTATTAAGCAATATCCTATGTCTCAGGAAGCTAGGCTTGGAATTAAACCTCATATTCAAAGACTTCTTG
ATCAAGGAATTTTGGTGCCTTGTCAATCACCTTGGAATACCCCTCTTTTACCTGTTAAAAAGCCTGGAACTAATGATTATAG
GCCAGTTCAGGATCTTAGAGAGGTTAATAAGAGAGTTGAAGATATTCATCCTACCGTTCCTAATCCTTACAACTTGCTTTCT
GGACTTCCTCCTTCTCATCAATGGTATACTGTGTTGGATCTTAAAGATGCTTTTTTTGTCTTAGATTGCATCCTACTTCTCA
ACCTCTTTTTGCTTTTGAATGGAGAGATCCTGAAATGGGAATTTCTGGTCAACTTACTTGGACTAGGCTTCCTCAAGGATTT
AAGAATAGTCCTACTTTGTTTAATGAAGCACTCCATAGAGATTTGGCTGATTTTAGAATCCAACATCCTGATCTTATTTTGCT
TCAATATGTTGATGATTTGCTTCTTGCTGCTACTTCTGAGTTGGATTGTCAGCAGGGTACTAGAGCTTTGCTTCAAACTCTT
GGAAATTTGGGATATAGAGCTTCTGCAAAGAAGGCTCAAATTTGTCAGAAGCAGGTTAAATATCTTGGATATTTGTTGAAGG
AGGGACAAAGGTGGTTGACTGAGGCTAGAAAAGAAACTGTTATGGGACAGCCAACACCAAAAACACCAAGACAACTTAG
GGAATTTCTTGGAAAGGCAGGATTTTGTAGACTTTTTATTCCTGGATTTGCTGAGATGGCTGCTCCTTTGTATCCTCTTACT
AAGCCAGGAACCCTCTTCAACTGGGGACCTGATCAGCAAAAGGCTTATCAGGAAATTAAGCAGGCACTTCTTACTGCTCC
TGCACTTGGACTTCCTGATCTTACTAAGCCTTTCGAACTTTTTGTGGATGAAAAACAAGGATATGCTAAGGGAGTTTTGACT
CAAAAGTTGGGACCTTGGAGAAGACCTGTTGCTTATCTTTCTAAGAAACTTGATCCTGTTGCAGCTGGTTGGCCTCCTTGT
CTTAGAATGGTTGCTGCTATTGCTGTTCTTACTAAGGATGCTGGTAAGCTCACTATGGGACAACCTCTTGTTATTCTCGCAC
CTCATGCTGTTGAAGCTCTTGTTAAGCAACCTCCTGATAGATGGCTTTCTAATGCTAGAATGACTCATTATCAAGCTCTTCT
TCTCGATACTGATAGGGTTCAATTTGGACCTGTTGTTGCTTTAAATCCTGCTACTCTTCTTCCTCTCCCTGAAGAAGGTCTT
CAACACAACTGTTTAGATATTCTTGCTGAGGCTCATGGAACTAGACCTGATCTTACCGATCAACCTCTTCCTGATGCTGATC
ACACTTGGTATACTGATGGTTCTTCTTTGCTTCAAGAAGGACAAAGAAAGGCTGGAGCTGCTGTTACTACCGAAACTGAA
GTTATATGGGCAAAAGCACTTCCTGCTGGAACTTCTGCTCAAAGAGCTGAATTGATTGCTCTTACACAAGCTCTTAAAATG
GCAGAAGGAAAGAAACTTAATGTTTATACTGATAGTAGGTATGCTTTTGCAACTGCTCATATTCATGGAGAAATTTACAGAAG
AAGGGGATGGTTGACTTCAGAAGGAAAGGAAATTAAGAATAAGGATGAAATCCTCGCTCTTTTGAAGGCTTTGTTTTTGCC
TAAGAGACTTTCTATTATTCATTGTCCTGGACACCAAAAGGGACATAGTGCAGAAGCTAGAGGAAATAGAATGGCTGATCA
AGCTGCTAGAAAGGCTGCTATTACTGAAACTCCAGATACTTCAACTCTTCTTATTGAAAATTCTTCTCCATCAGGAGGATCT
AAGAGGACAGCTGATGGATCTGAATTTGAA

4. Sequences of PBS and RT templates used in this study

Target sites	sgRNA	PBS	RT templates
Cm^R H139Y	GACATGGAAGCCATCACAAA	GTGATGGCTTCCA	CATCATGCCGTTT
PDS3-1	CCTCCAGATAGCTGCATGGA	ATGCAGCTATCTG	TCATCCTACC
PDS3-2	TTCATGTTCTTGTTGGAAGT	TCCAACAAGAACATG	TCGGATAAGCACTGACT
ALS	TGGTTATGCAATGGGAAGAT	TTCCCATTGCATAAC	TGTAGAACCGACC

5. Primers used in amplicon

Prime Name	Sequence
Cm-F1	TTATCCCCTGATTCTGTGGATAACCG
Cm-R1	TTCCATGAGCAAACTGAAACGTTTTCAT
SDCX-F1	TCAGTCTTGTGCTACACACCGC
SDCX-R1	CGGATAAGCACCGACTTCCAACAAG
SDCX-F2	TGCATGGAAGGATGAAGATGGGG
SDCX-R2	CAGCTGACTAGCAAGTAAGAATATGCG
ALS-F1	GGCGCAGTTCTACAATTACAAGAAACCA
ALS-R1	CCATTCGGGATCATCGGCAAC
ED-PDS1-F2	AGAGTAGTTTACTGGAAGATATCTTTTGGATATATATTTTGTTAAGT
ED-PDS1-F2	TAACCAGTGTTTTGCAACTTTCTTCAAATTGAT
ED-PDS2-F1	TCCAAAACTCATACCCTCTCTG
ED-PDS2-R1	CCATTTAAGGGTGCTGGTAG
ED-ALS-F1	CCTGCTGCGATTGGAGCG
ED-ALS-R1	CCAACAGGTAAGGTCCTGGTGT
